# Co‐Creation Study Protocol for Developing a Nurse‐Led Intervention to Deprescribe Benzodiazepines and Z‐Hypnotics in Primary Care Aimed at Empowering Women

**DOI:** 10.1111/hex.70361

**Published:** 2025-09-18

**Authors:** Amaia Cuesta‐Zigorraga, Carlota Las Hayas Rodríguez

**Affiliations:** ^1^ Nursing Department, Faculty of Health Sciences University of Deusto San Sebastian Spain; ^2^ Department of Medicine, Faculty of Health Sciences University of Deusto Bilbao Spain; ^3^ Network for Research on Chronicity Primary Care and Health Promotion (RICAPPS) Bilbao Spain

**Keywords:** deprescribing, empowerment, nursing leadership, patient participation, primary healthcare

## Abstract

**Background:**

Gender inequality in mental health is a persistent challenge that requires innovative solutions for its resolution. Interventions in primary care can be useful in narrowing the gap in the consumption of psychotropic drugs.

**Objective:**

This study proposes female empowerment as a non‐medical alternative for facing women's daily lives, which are often pathologised, and aims to develop a group intervention in primary care that promotes female empowerment and reduces the use of benzodiazepines and Z‐hypnotics (BDZ/ZH).

**Methods:**

Co‐creation is presented as a novel methodology for developing health interventions. Participatory workshops will be organised involving women who use BDZ/ZH and health professionals, including family doctors, primary care pharmacists, and nurses. These workshops will collectively identify the needs of participants and contribute to the design of the intervention, which will be developed as a nurse‐led initiative.

**Discussion:**

This methodology is essential for designing interventions that respond effectively to the particularities of women who use BDZ/ZH and is valuable in adapting to the actual clinical context of the health professionals involved in their care.

**Conclusion:**

Nurses' ongoing patient relationships, deep knowledge of individual life contexts, and expertise in health promotion and education make them especially suited to lead empowerment‐based interventions.

**Patient or Public Contribution:**

This study protocol outlines a co‐creation process involving patients and healthcare professionals from diverse fields of primary care—pharmacists, family physicians and nurses—to collaboratively design an intervention aimed at empowering women as a strategy for discontinuing benzodiazepines (BDZ) and hypnotics. The goal is to leverage both the lived experiences of patients and the clinical expertise of healthcare providers to develop a tailored, effective intervention. In the future implementation of the intervention, nurses are expected to take a leading role.

**Clinical Trial Registration:** This manuscript outlines a protocol for a participatory process and, as such, it has not been registered as a clinical trial.

## Graphical abstract

xxxx

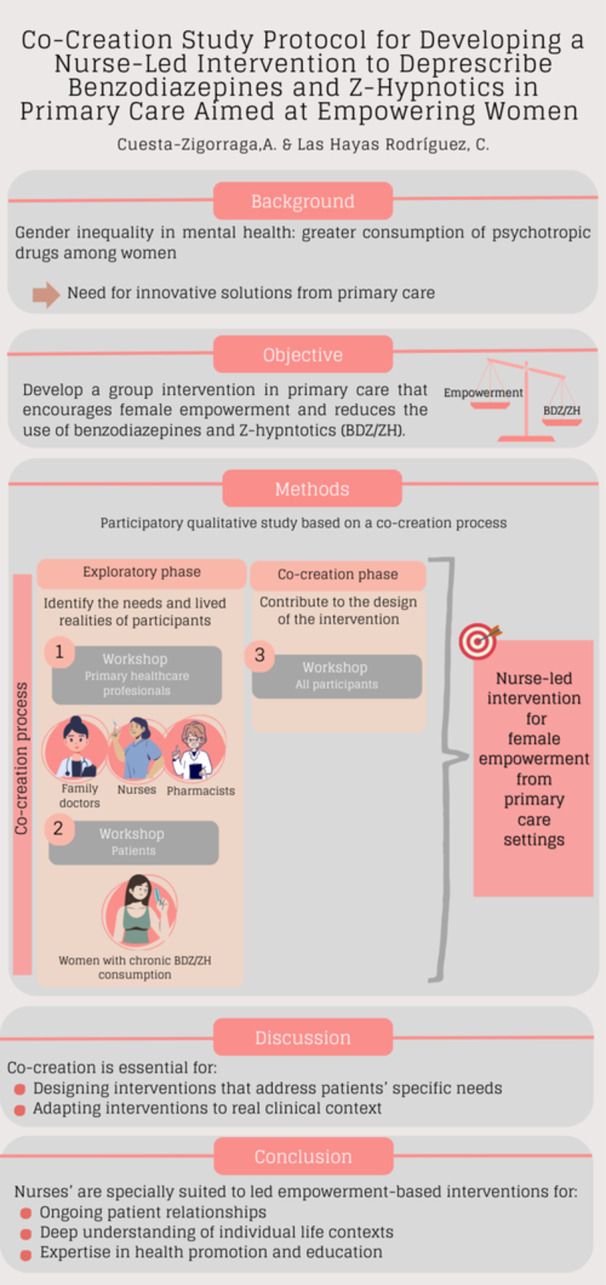

**Abbreviations**
BDZ: benzodiazepines
SO: specific objectives
ZH: Z‐hypnotics

## 1 Introduction

Evolutionary data show an uninterrupted increase in the consumption of anxiolytics and hypnotics in recent years [1, 2]. Women receive twice as many diagnoses of anxiety and depression and consume twice as many psychotropic drugs as men. Gender inequalities, defined as the systematic disadvantage experienced by individuals based on their gender, are evident in the field of mental health, with women facing a higher medicalisation of their mental health issues compared to men [3]. Such inequalities in the field of mental health represent a persistent challenge that requires innovative and collaborative solution approaches.

The co‐creation methodology allows the use of available knowledge in a potentially effective way to address complex problems in the healthcare system [4]. One of these issues is gender inequity in mental health and the use of psychotropic drugs. This can be addressed not only by finding innovative solutions but also by involving women—who are the primary users of these medications—in the design of strategies to tackle the problem. Co‐creation challenges patient passivity and focuses on the individual's needs, involving all agents concerned with the construction of knowledge in an egalitarian manner [5]. In this sense, co‐creation not only seeks to solve problems but also serves as a tool for the personal empowerment of those involved, transferring to them the agency of their own health and the healthcare interventions that affect their well‐being. Patient empowerment is acknowledged as a crucial element in enhancing health outcomes, improving communication between patients and healthcare professionals, increasing treatment adherence, and ensuring the efficient use of primary healthcare resources [6].

## 2 Background

The intersection of structural social inequalities and gender bias in clinical practice produces notable differences between genders. These differences affect both the diagnostic process and therapeutic interventions. Female experiences of everyday life are often pathologised and treated predominantly with psychopharmaceuticals. It has been shown that in cases of equal symptoms of anxiety and depression, women are more likely than men to receive such a diagnosis [7, 8] and are consequently treated with psychopharmaceuticals [9]. In contrast, the male gender is associated with an underdiagnosis of emotional disorders, such as depression [10]. Specifically, in the Basque Country (Spain), 17.1% of women report poor mental health, compared to 10.0% of men. Moreover, women are more frequently diagnosed with depression or anxiety (5.8% vs. 2.1%), and their use of psychotropic drugs is significantly higher (12.5% vs. 4.9%) [3].

In addressing gender inequalities in mental health, women's empowerment can be an effective mitigating strategy for these inequities. The theoretical rationale for women's empowerment focuses on recognising existing gender inequalities in society. The term empowerment was coined in 1995 at the Beijing World Conference on Women and refers to ‘increasing women's participation in decision‐making processes and access to power’ [11]. Today, it also involves another dimension: the awareness of the power that women possess, individually or collectively. In addition, it is related to the acquisition of dignity as a person. Empowerment is therefore a dynamic process of identity construction that includes the acquisition of power, control over one's life, and the ability to make choices [12]. In this sense, empowerment can facilitate the withdrawal of psychotropic drugs. This allows women to take an active role in managing their health, questioning the use of drugs, and opting for non‐medicated alternatives to face the challenges of everyday life.

Nurses have led interventions aimed at promoting patient empowerment across various healthcare contexts. These nurse‐led initiatives have been shown to be effective in areas such as hypertension management [13, 14], during discharge from the intensive care unit [15], gynaecologic oncology chemotherapy [16], early rheumatoid arthritis [17] and breast cancer processes. Education, psychosocial support, promotion of self‐care, and coping strategies are the unifying keys to nurse‐guided empowerment interventions [18]. However, it is important to note that these initiatives have not specifically addressed female empowerment.

Nurses have also been involved in medication deprescription by supporting dose reduction, discontinuation of medications, and transition to safer alternatives [19]. Additionally, nurses have experience in leading interventions focused on the deprescription of BDZ, achieving high levels of abstinence: 80% at 6 months and 65% at 1 year [20].

The design of public health policies and health interventions has traditionally followed a top‐down approach. However, there is growing interest in repositioning patient power and shifting decision‐making capacity. Knowledge and power mobilisation are ways to bridge the gap between theory and practice [21]. Health interventions, particularly those aimed at empowerment, can be enriched by the application of participatory methodologies in their creation. The co‐creation methodology facilitates the detection of the patient's empowerment needs, allows the adaptation of the intervention's content and promotes the design of interventions centred on the end user [22].

Co‐creation is a participatory methodology in which knowledge is generated collaboratively between academics and other stakeholders [23]. Therefore, in co‐creation, the knowledge and experience of patients are valued alongside those of professionals, with equal, mutual, and reciprocal relationships between them. People and their experiences are recognised as assets with the aim of harnessing the capabilities of individuals for a mutually beneficial relationship [24, 25, 26].

Numerous end‐user benefits of collaboration in this participatory process have been identified. The co‐creation of interventions can enhance immediate individual health outcomes by improving the skills needed to manage medical situations. Specifically, positive outcomes are observed in the emotional health of end users [27, 28]. This increases confidence in everyday life and creates a sense of pride and accomplishment among participants [29, 30]. In addition, the positive impact on the healthcare system is highlighted, as co‐creation promotes self‐efficacy, improves access to health services and strengthens community relationships [27].

This article describes the protocol for co‐designing a group intervention for women's empowerment in collaboration with primary care health professionals, patients and researchers. The resulting intervention will be nurse‐led in primary care settings.

### 2.1 The Study

#### 2.1.1 Objectives and Hypothesis

The objective of this study is to co‐create a nurse‐led group intervention for women aimed at promoting female empowerment and reducing the consumption of BDZ and Z‐hypnotics (BDZ/ZH) in an effective way at the primary care level.

Co‐creation involves all relevant stakeholders in a process where the knowledge and experiences of participants are equally valued. This approach fosters mutual, reciprocal and equitable relationships. It promotes collaborative cooperation throughout every stage, from problem definition to the design of solutions, ensuring active involvement from all parties [31].

In this regard, the intervention will be designed in collaboration with the patients and the healthcare professionals who care for them, such as primary care pharmacists, family physicians and nurses. In addition, the study aims to create an implementation training programme for nurses to ensure the success and sustainability of the intervention.

Therefore, the following hypothesis is proposed: The application of the co‐creation methodology, with the active involvement of key stakeholders (patients and healthcare professionals) working collaboratively to address BDZ/ZH consumption in primary care, will result in an effective intervention that empowers women and facilitates the deprescription of these drugs.

## 3 Methodology

### 3.1 Study Design

A co‐creation method is proposed for designing this intervention with the collaboration of primary healthcare professionals, patients and a technical team. Co‐creation is a Participatory Action Research (PAR) method that involves stakeholders in all stages of the research process to collaboratively identify problems, generate solutions and implement actions that are contextually relevant and socially meaningful.

The co‐creation methodology has been chosen because it focuses on the experiences of stakeholders, promoting direct interaction among them and involving all relevant stakeholders in the process (in this case, patients and healthcare professionals). On the other hand, and in contrast, co‐design focuses on specific concerns related to the design of products or services, where users actively collaborate in creating and improving them, but with a more limited focus compared to co‐creation. Finally, co‐production focuses on the collaboration among stakeholders to produce a good or service, where participants play a more passive role, primarily in the implementation of the intervention or in producing pre‐defined outcomes [31].

A total of three co‐creation workshops are planned: the first two will explore the experiences and needs of health professionals and patients, followed by a collaborative workshop to co‐create the intervention. During the co‐creation workshops, the technical team will propose exercises to facilitate active participation, explore the participants' perceptions and collaboratively describe the characteristics and content of the intervention.

The specific objectives (SO) of each session and the proposed dynamics are described in detail below.

PAR systematises and analyses the information collected from the interaction of the stakeholders involved. These techniques are focused on understanding the experiences, perceptions and opinions of the participants. During participatory dynamics, certain monitoring indicators will be measured through direct observation. These indicators will include the interaction of the participants in each proposed dynamic, the degree of consensus and the relevance of the contributions in shaping the structure and content of the intervention.

The co‐creation process will also be evaluated quantitatively through a satisfaction survey completed by the co‐creation participants. Further analysis will be conducted to determine whether the intervention reflects the co‐creators' preferences and needs. The survey will evaluate the quality of the information received, the methodology used, the equity in participation, and the perceived usefulness of the co‐created intervention in promoting women's empowerment or reducing the use of BDZ/HZ. The anonymised self‐reported survey will include a Likert scale with five response options ranging from ‘totally agree’ to ‘totally disagree’ to gauge satisfaction. Socio‐demographic data will also be collected through the questionnaire, including participants' educational level, economic status, nationality, age, type of employment and marital status.

To ensure the co‐creation process meets quality standards and objectives, a methodology specialist will conduct an audit of the process. In addition, the Guidance for Reporting Involvement of Patients and the Public checklist (GRIPP2) [32] will be used to ensure that patient and public involvement (PPI) is based on the best available evidence. The GRIPP2 checklist allows systematic documentation of key aspects of PPI, including objectives, methods and impact, which improves the quality and transparency of research reporting, thus facilitating replication.

**Figure 1:**
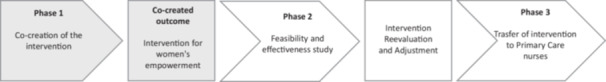
Summary of the research process.

The co‐creation phase is the first of a larger project with two additional phases. Once the intervention has been co‐created with the key agents, its feasibility and effectiveness will be tested in a future project. Finally, the intervention will be transferred to the nursing collective for replication in different health centres (see Figure 1).

#### 3.1.1 Study Setting and Participants

The participants in the co‐creation process are described below.

1. End users—women with chronic BDZ/ZH use—who are linked to the Osakidetza Primary Care Network, Hernani Health Center (Gipuzkoa Health Area, Basque Country, Spain). Because of the varying contexts of this problem and the need to apply an intersectional view, the women contributors in this study belong to different social division categories in terms of age, socio‐economic position, educational level and marital status. A cohort of 7–10 participants will be recruited to provide information on their specific needs.
2. Healthcare professionals (primary care pharmacists, family physicians and community nurses), 7–10 of whom will participate in this study. A minimum of two pharmacists, two family physicians and three nurses will be recruited. The health professionals participating in the intervention co‐creation workshops are from the same health centre as the patients involved. This horizontal approach narrows the distance between professionals and patients, promoting more open and collaborative communication regarding health interventions. In addition, the inclusion of health professionals guarantees a solid clinical perspective appropriate to the real clinical context.
3. The technical team, whose main function is to ensure the correct execution of the methodological aspects of the study and to document the relevant information from the co‐creation workshops. The technical team is also responsible for achieving the SO of the co‐creation workshops through collaborative dynamics. It is composed of research staff and a specialist in participatory methodologies. The research staff, consisting of a family doctor, a pharmacist and a nurse, who match the profiles of the participating health professionals, will take detailed notes by direct observation during the workshops to record the interactions and agreements reached. This composition seeks not only to facilitate a more contextualised understanding of the participants' experiences but also to promote an interdisciplinary perspective that enriches data interpretation. The methodology specialist will audit the methodological process to ensure that it adheres to quality standards and objectives. Regarding sample size, participatory methodologies such as co‐creation with patient involvement require approaches adapted to the particularities of these collaborative processes. In co‐creation, the focus is usually not on statistical representativeness, but on the depth of participation and the diversity of perspectives. Likewise, the operational viability of the participatory process and the dynamics proposed in the sessions limit the sample size to a manageable number.

#### 3.1.2 Eligibility Criteria

Table 1 shows the inclusion and exclusion criteria adapted to the demographic and clinical characteristics of this study.

**Table 1:** Study inclusion and exclusion criteria.

Inclusion criteria	Exclusion criteria
Patients	
– Subjects must be over 18 years of age.	– Epilepsy
– Sex—subjects whose biological sex is female are selected for this study.	– Dementia
– Subjects must have used benzodiazepines or Z‐hypnotics for more than 3 months.	– Palliative care patients
	– Open episode in the Mental Health Network
	– Severe psychiatric comorbidity with external follow‐up in the Mental Health Network
	– Severe psychiatric comorbidity without follow‐up in the Mental Health Network
	– Failure to sign the informed consent form
	– Not fluent in the language in which the intervention will be offered (Spanish)
Healthcare professionals	
– The staff must be actively involved in patient care at the participating centre.	– Existence of conflicts of interest
– Participants must have a professional profile in family and community medicine, nursing, or primary care pharmacy	– Failure to sign the informed consent form

#### 3.1.3 Procedure for Co‐Creating the Intervention

A total of three co‐creation workshops are planned. The first two workshops are exploratory in nature, and the last workshop is for collaborative evaluation of the co‐creative intervention.

In the exploratory stage, a preliminary data analysis is planned to involve each specific group, starting with the health professionals and then with the patients, to understand their experiences and needs.

The contributions of the participants will then be incorporated by adjusting Aróstegui and Martínez's [33] intervention design for relapse prevention from a gender perspective. The central axis of Aróstegui and Martínez's [33] work is gender inequality in the establishment and cessation of psychotropic drug use, and this is the same axis on which this study is based. The cited work should be consulted for more detailed intervention information. The proposed guide serves here as a flexible starting point; its content and structure will be adapted and aligned with the perspective of the people involved in the co‐creative process. Similarly, the content of the intervention will be adjusted and focused on the characteristics and needs of the target population.

Intervention Mapping is used to plan interventions that are effective, contextualised and aligned with the real needs of the target population. Co‐creating based on an existing intervention ensures that the resulting intervention retains its theoretical base and evidence but is enriched and personalised by the inclusion of the voices and experiences of all actors involved. In this way, the effectiveness and feasibility of its future implementation are increased [34].

The last workshop focuses on developing the content and structure of the intervention. The group intervention prototype produced after incorporating the participant contributions from the exploratory workshops will be re‐evaluated. All participants—health professionals and patients—will attend the last workshop. During this meeting, the participants will jointly evaluate the content and structure of the provisional version of the intervention. They will assess whether the presented intervention structure is sustainable in clinical practice and whether it fits the needs of the end users. In addition, the effectiveness of the objectives and content of the intervention for the women's empowerment process will be evaluated. Data gathered during the last workshop will be analysed to develop the content and structure of the final intervention. Table 2 describes the participants and SO of each co‐creation workshop.

**Table 2:** Participants and SO of the co‐creation workshops.

Variable		Co‐creation workshop with primary healthcare professionals	Co‐creation workshop with patients	Co‐creation workshop with all participants
Participants		Pharmacists Family physicians Nurses	Women with chronic use of benzodiazepines and/or Z‐hypnotics	Healthcare professionals Patients
SO	Pharmacological regimen	Detect collectively and from different health fields (pharmacy, family medicine and nursing) the usual primary care interventions aimed at the withdrawal of potentially inappropriate prescriptions of BDZ/ZH.	Analyse the patient's perception and attitude toward her pharmacological BDZ/ZH regimen. Explore knowledge of clinical indications for prescription withdrawal with BDZ/ZH.	Agree on the prioritisation of the objectives and the relevance of the issues to be addressed in the intervention prototype. Evaluate the appropriateness of the dynamics and format of the prototype intervention sessions.
	Empowerment	Collectively define the content of a group intervention for women's empowerment.		
	Nurse‐led group intervention in primary care	Collectively determine the characteristics of temporality, sustainability and structure of a group intervention led by primary care nurses to facilitate its implementation.	Collectively determine the characteristics of temporality, sustainability and structure of a group intervention led by primary care nurses to facilitate patient adherence.	

Abbreviations: BDZ/ZH = benzodiazepines and Z‐hypnotics, SO = specific objectives.

The co‐creation process has a multidisciplinary identity, where patients and healthcare professionals collaborate in a shared space to assess issues and contribute to their viewpoints. The technical team will be responsible for promoting equitable participation through the dynamics described below, to gather valuable information about participants' experiences and needs, as well as about insights to co‐create the intervention. This technical team also has a multidisciplinary nature, including the same profile of health professionals who participate in the co‐creation of the intervention (a pharmacist, a family physician and a nurse). Therefore, each participant is expected to attend two workshops. Each workshop is estimated to last 1 h and 30 min. The co‐creation workshops will be held at the Casa de Cultura of Hernani, located 200 m from the participating health centre. This is aimed at providing a neutral venue and fostering an equitable environment to ensure that health professional/patient power dynamics do not interfere with the workshop processes.

The three co‐creation workshops will take place over a period of 2 months. After the first workshop with healthcare professionals, 1 month will be dedicated to recruiting patients who wish to participate. Subsequently, the second workshop will be conducted with patients, and the third workshop will include all participants (healthcare professionals and patients), with a 1‐week interval between them.

#### 3.1.4 Dynamics and Exercises Applied in the Co‐Creation Workshop

The co‐creation methodology consists of collaborative group dynamics among the participants in order for them to design the intervention together. The co‐creation workshops address the needs and key issues for patients and health professionals, integrating their perspectives into the design, content and structure of the intervention.

Each proposed participatory dynamic is designed to respond to the SO defined for each group of participants and workshops (see Table 2).

a. **Objective:** Collective detection and analysis of real clinical interventions for BDZ/ZH discontinuance in primary care.

In the first phase—the exploratory phase—the actual aspects related to the pharmacological regimen will be analysed using the *journey mapping* technique. This tool consists of discussing in small groups and then capturing the answers to the triggering questions posed by the technical team in a collaborative conceptual map.

**Figure 2:**
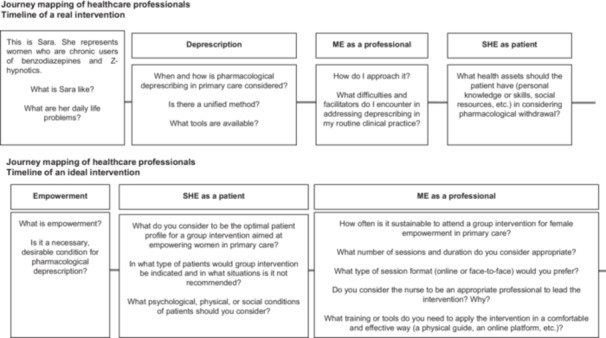
Journey mapping with healthcare professionals in the exploratory phase.

**Figure 3:**
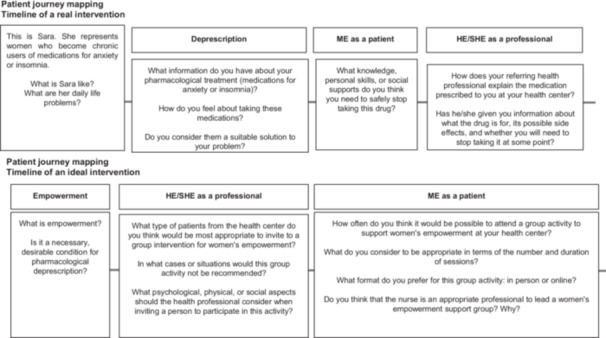
Journey mapping with patients in the exploratory phase.

- In the health professional group, the study seeks to explore the usual interventions regarding BDZ/ZH withdrawal in primary care clinical practice. The aim is to identify the tools available and the facilitators and barriers to implementing interventions for the withdrawal of potentially inappropriate prescriptions. For this purpose, a fictitious clinical case (in which the situation of an individual with chronic BDZ/ZH consumption and the daily problems she faces is explored) acts as a starting point.
- In the patient group, their relationships with their pharmacological regimen are examined together with their knowledge of the withdrawal process. Triggering questions are used to determine how the patient perceives and manages her treatment, what support she considers necessary to stop, and how she has been oriented by her health professional regarding the use of medication. Here, the starting point is a fictitious case representing women who chronically use medication for anxiety or insomnia and exploring their characteristics and the problems they face daily. The language used in communicating with patients is adapted to their level of understanding to ensure that information is clear and accessible.

b. **Objective:** Collective definition of the content and key characteristics of an ideal nursing‐led group intervention in primary care aimed at women's empowerment.
  In the second part of the workshops, held exclusively with each group, the characteristics of an ideal group intervention are collectively defined. This intervention will be oriented towards female empowerment and will be led by primary care nurses. For this purpose, the *journey mapping* technique is applied again, using key questions to unearth information and jointly elaborate a conceptual map. The objective is to evaluate how empowerment can be a key factor in medication withdrawal and what characteristics patients should have to benefit from a group intervention. This study also seeks to determine preferences regarding the format, frequency and leadership of this intervention, specifically assessing nursing leadership.
  Figure 2 presents the journey mapping with healthcare professionals during the exploratory phase, namely in the co‐creation workshop conducted exclusively with them. The journey mapping applied in the workshop with patients is shown in Figure 3.
c. **Objective:** Feedback on the prototype intervention.

Finally, in the last workshop, attended by all participants, two group dynamics were developed with the objective of receiving feedback on the group intervention prototype presented, following Aróstegui and Martínez's [33] design.

1. ***Priority matrix:*** Participants discuss in small groups and reach consensus on the prioritisation of objectives and the relevance of the prototype intervention topics. In this context, the participants are tasked with distributing scores according to the relevance of working in each area within an intervention that promotes female empowerment aimed at the withdrawal of BDZ/ZH. Specifically, the participants evaluate the key areas worked on in the prototype intervention: self‐esteem, self‐image, pharmacological treatment, gender‐based violence, couple relationships, sexuality, motherhood and self‐care.
2. ***Idea laboratory:*** In small groups, participants assess the content of the prototype, providing constructive feedback for each session using a short questionnaire. This questionnaire analyses, on the one hand, the appropriateness of the proposed exercise in relation to the session's objectives. A numerical rating of 1–10 is used, complemented by a scale of smiley faces to visually represent the level of satisfaction. The questionnaire also evaluates whether the structure and format of the session are appropriate. This is explored through an open‐ended question, in which participants are asked to elaborate on their responses.

The detailed plan for each workshop is listed in Table 3.

**Table 3:** Planning and dynamics of the co‐creation workshops.

Duration	Co‐creation workshop with healthcare professionals	Co‐creation workshop with patients	Co‐creation workshop with all participants
10 min	Welcoming the participants and contextualisation		
	Presentation of meeting objectives and session plans		
30 min	**Journey mapping**	**Journey mapping**	**Priority matrix**
	**Timeline of a real intervention**	**Timeline of a real intervention**	Participants discuss in small groups and reach consensus on the prioritisation of objectives and the relevance of the issues to be addressed in the prototype intervention by assigning a score based on importance.
	Participants are divided into small groups with representatives from different health profiles to explore common clinical practices for deprescribing BDZ/ZH. Subsequently, the contributions will be pooled and reflected in a collaborative conceptual map.	Participants reflect in small groups their perceptions and attitudes towards the pharmacological guidelines and explore their knowledge about the deprescription of BDZ/ZH. Subsequently, the contributions will be shared and reviewed in a collaborative conceptual map.	
10 min	Pause		
40 min	**Journey mapping**		**Idea laboratory**
	**Timeline of an ideal intervention**		Participants evaluate the content of the prototype intervention in small groups. They provide constructive feedback using a brief questionnaire for each intervention session evaluated. The adequacy of the dynamics and their format are analysed.
	Participants are divided into small groups to discuss the characteristics (content and structure) of an ideal nurse‐led group intervention for female empowerment in primary care. Subsequently, the contributions will be pooled and reflected in a conceptual map.		

Abbreviation: BDZ/ZH = benzodiazepines and Z‐hypnotics.

#### 3.1.5 Data Collection and Analysis

Data will be collected using a mixed‐methods approach. In accordance with the principles of PAR, data will be collected through direct observation and participatory workshops, enabling joint reflection, co‐creation of knowledge and iterative evaluation of the process. Inductive thematic analyses will be applied to the data collected from the co‐creation workshops [35]. Two sources of data will be used: structured templates completed during the workshop exercises and the notes taken by the technical team throughout the sessions. The inductive approach will allow organic themes to emerge from the data, while the deductive approach will apply pre‐established frameworks to guide the analysis. Quantitative data will be collected with the satisfaction survey at the end of the process. This data will be processed using descriptive statistics, including measures of central tendency (mean, median) and frequency distributions.

#### 3.1.6 Recruitment Procedure and Ethical Considerations for Each Type of Participant

##### 3.1.6.1 Recruitment Procedure

a. Health professionals—primary care pharmacists, family physicians and nurses—who are interested in participating are identified in collaboration with the healthcare service providers involved. Health professionals are invited by the research staff to collaborate in the participatory process. Those professionals who meet the eligibility criteria and are interested in the project are invited to participate in the study. The objectives and the procedure for collecting and signing informed consent are communicated to them.
b. End users who are women with chronic BDZ/ZH consumption are invited by family physicians or nursing staff to join the participatory co‐creation process after favourable evaluations based on the eligibility criteria. Patients are recruited by their referring nurses or family physicians during other scheduled consultations within the previously agreed recruitment dates. Health personnel exclusively deal with the patient's personal data, a practice that is permitted in their usual care. Thus, health personnel ensure diversity and pluralism by selecting participants from different social categories in terms of age, socio‐economic status, educational level and marital status. There is no stipulated sample size for each social category. The selection guideline is that the recruited sample should be representative of women who use BDZ/ZH.
  Information regarding the co‐creation project and details on how to participate are provided to the patient by the health personnel orally and in writing on an information sheet. This includes the description of the study, the risks and benefits, and the confidentiality protocol. Signing the informed consent document verifies that the patient has received and understood the information provided by the health professional and voluntarily agrees to participate in the co‐creation process of the group intervention for women's empowerment.
c. The technical team, led by the principal investigator of the study, is responsible for distributing the physical information sheets and collecting consent signatures.

##### 3.1.6.2 Ethical Considerations

This study project has been approved by the Research Ethics Committee of the Gipuzkoa Health Area in accordance with Law 14/2007 on Biomedical Research, the ethical principles of the Declaration of Helsinki, and other applicable principles.

This study involves the processing of personal data; therefore, the researchers always guarantee confidentiality in data handling. This follows the regulations on the protection of personal data, particularly the European Regulation 2016/679 of April 27 on general data protection and the Organic Law 3/2018 of December 5 on the Protection of Personal Data and Guarantee of Digital Rights.

The collection and analysis of data during the co‐creation process will be conducted in a rigorous and ethical manner, respecting the confidentiality and privacy of the people involved. Throughout the co‐creation process, the research staff will preserve the anonymity of the participants, including in field notes, written reports and other publications resulting from this study.

The personal and questionnaire data are stored in separate databases. The questionnaire database does not contain any identifiers linking to the individual. Once the data have been dissociated, they are stored in a secure, access‐controlled manner, available only to authorised research personnel. All statistical analyses will be performed using exclusively anonymised data, and under no circumstances will data that allow the identification of the individual be revealed, ensuring the confidentiality, privacy and security of the information.

## 4 Discussion

The participation of patients and healthcare personnel in health intervention processes is beneficial to clinical practice and the parties involved. Co‐creation plays a key role in transforming healthcare towards a more collaborative and value‐centred model [36]. In the same way, co‐creation enhances the emotional well‐being of participants, fosters self‐efficacy and strengthens both service access and community ties [27, 28, 29, 30]. All in all, the application of PAR in public health generates shared value and the integration of diverse knowledge. This improves the relevance, sustainability and effectiveness of interventions [31].

This study introduces an innovative approach to deprescription by actively incorporating both patients and healthcare professionals. This element is not typically present in current deprescription practices in primary care settings, which tend to be more clinician‐led and less participatory [37].

Using sex assignment at birth as a criterion for inclusion in the study may be a limitation, as it excludes individuals whose gender identities do not correspond to their assigned sex at birth. It is important to consider people with diverse experiences, including those who identify as female and comply with the female gender. The current health registry is based on a sex dichotomy, which makes it difficult to access a complete sample based on the proposed methodology. In relation to this, the absence of comparison with the male population in the design phase could limit the identification of gender‐specific factors. Including the female voice in the co‐creation of an intervention for women is essential to ensure that their needs, experiences and priorities are reflected, but on its own it is not enough: excluding the male perspective leaves out the role that men play (as partners, caregivers or agents of social change) and deprives the design of truly comprehensive and sustainable solutions in the family and community environment.

A small sample is used for this study because the co‐creation methodology requires in‐depth interaction with the participants and the fostering of a collaborative environment. In addition, the sample size was selected to ensure effective dynamics during the co‐creation workshops and to align with the sample sizes used in similar previous studies within the same field [38, 39].

The process by which family physicians or referring nurses at health centres select patients for participation in the proposed study can result in the exercise of asymmetrical power relationships, which can be transferred to the dynamics of co‐creation. It is vital to recognise this dynamic and the need for healthcare professionals to provide the necessary information to patients to facilitate informed decision‐making free of undue pressure. Addressing the power dynamics in co‐creation by facilitating an inclusive environment fosters a process that promotes equal participant collaboration. This power imbalance can also be transferred to participatory sessions, producing a dominance bias due to the existence of voices with higher status, potentially limiting the diversity of contributions.

Additionally, co‐creation methodology may involve a selection bias, as it tends to include stakeholders who are motivated by the process and may exclude less active but equally relevant voices.

Finally, the social desirability bias must be taken into consideration during co‐creation sessions due to the possible tendency of participants to offer answers that they consider socially acceptable or that please the researchers. This bias can be minimised by implementing neutral facilitation and guaranteeing the confidentiality of their participation. In this sense, the beliefs or style of the facilitator can influence how discussions develop or what topics are prioritised.

## 5 Conclusion

This project addresses the phenomenon of the medicalisation of women's discomfort at its roots by providing women with strategies to cope with everyday life. Women's empowerment is a key means of providing the necessary tools and support to avoid resorting to or depending on pharmacotherapy.

Co‐creating the intervention for women's empowerment with the agents involved in this phenomenon will contribute to scientific and practical knowledge for addressing the problem of BDZ/ZH use in the female population at the primary care level.

The essence of this co‐creation study lies in the integration of diverse perspectives from various healthcare professions within the primary care setting, as well as from both patients and healthcare providers. It is through this collaborative exchange that the true value of co‐creation is realised.

The role of nurses is fundamental in the leadership of these interventions, as they are in a privileged position due to their continuous contact with and knowledge of patients' life histories. In addition, nurses are experts in health promotion and education for the self‐management of health by adopting a community approach in their practice.

The social and salutogenic approaches discussed in this study, based on health assets, can have significant impacts on the integral aspects of women's primary healthcare. This study also contributes to addressing gender inequities in mental health and promoting pharmacological withdrawal and care strategies sensitive to individual needs. Considering the experiences and perspectives of end users and health personnel in the design process ensures that the intervention is relevant, effective and focused on the real needs of the parties involved.

## Permission to Reproduce Material From Other Sources

No material from other sources has been reproduced in this manuscript.

## Author Contributions

**Amaia Cuesta‐Zigorraga:** conceptualisation, writing – original draft, methodology. **Carlota Las Hayas Rodríguez:** writing – review and editing, methodology, supervision.

## Ethics Statement

This study project has been approved by the Research Ethics Committee of the Gipuzkoa Health Area in accordance with Law 14/2007 on Biomedical Research, the ethical principles of the Declaration of Helsinki, and other applicable principles (Code: CUE‐EMP‐2024‐01).

## Consent

Informed consent was obtained from all subjects involved in the study.

## Conflicts of Interest

The authors declare no conflicts of interest.

## Supporting information

Study protocol Visual abstract.

## Data Availability

The data that support the findings of this study are available from the corresponding author upon reasonable request. This statement is not applicable as this manuscript is a study protocol, and no data have been collected or analysed at this stage.
