# Supplementary figures and images for: Co‐Creation Study Protocol for Developing a Nurse‐Led Intervention to Deprescribe Benzodiazepines and Z‐Hypnotics in Primary Care Aimed at Empowering Women
Source: Health Expect. 2025 Sep 18;28(5):e70361. doi: 10.1111/hex.70361 (PMC12445199; doi:10.1111/hex.70361)

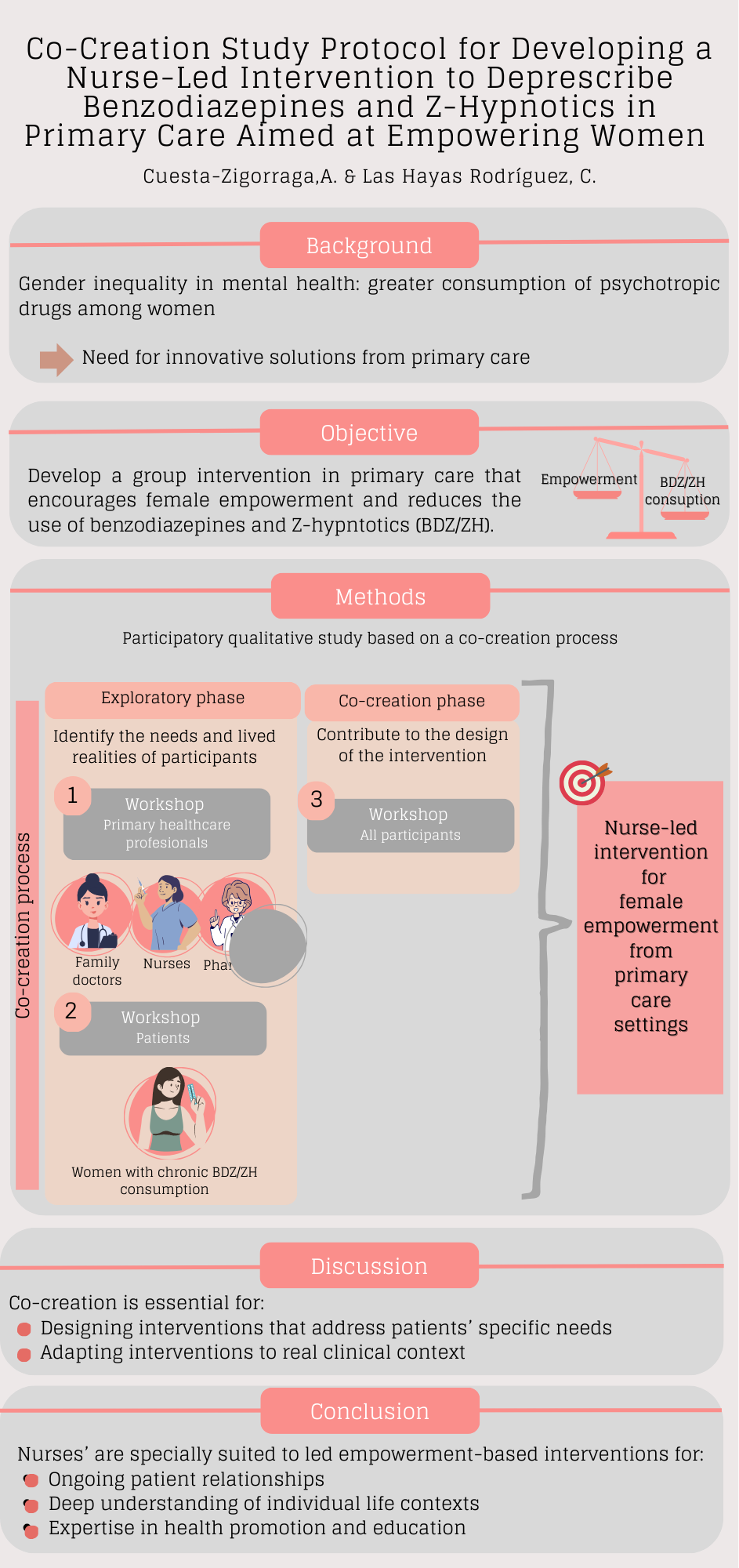

Supplement: Supplementary file 1 — Study protocol Visual abstract. [file HEX-28-e70361-s001.png]
